# A Cross-Cultural Examination of SNS Usage Intensity and Managing Interpersonal Relationships Online: The Role of Culture and the Autonomous-Related Self-Construal

**DOI:** 10.3389/fpsyg.2016.00376

**Published:** 2016-04-13

**Authors:** Soon Li Lee, Jung-Ae Kim, Karen Jennifer Golden, Jae-Hwi Kim, Miriam Sang-Ah Park

**Affiliations:** ^1^Jeffrey Cheah School of Medicine and Health Sciences, Monash University MalaysiaBandar Sunway, Malaysia; ^2^Halal Ecosystem (HE) Multidisciplinary Research Platform, Monash University MalaysiaBandar Sunway, Malaysia; ^3^Department of Psychology, Chung-Ang UniversitySeoul, South Korea

**Keywords:** culture, cross-cultural comparison, self-construal, social networking sites, social networking sites use

## Abstract

Perception of the autonomy and relatedness of the self may be influenced by one's experiences and social expectations within a particular cultural setting. The present research examined the role of culture and the Autonomous-Related self-construal in predicting for different aspects of Social Networking Sites (SNS) usage in three Asian countries, especially focusing on those aspects serving interpersonal goals. Participants in this cross-cultural study included 305 university students from Malaysia (*n* = 105), South Korea (*n* = 113), and China (*n* = 87). The study explored specific social and interpersonal behaviors on SNS, such as browsing the contacts' profiles, checking for updates, and improving contact with SNS contacts, as well as the intensity of SNS use, hypothesizing that those with high intensity of use in the Asian context may be doing so to achieve the social goal of maintaining contact and keeping updated with friends. Two scales measuring activities on other users' profiles and contact with friends' profiles were developed and validated. As predicted, some cross-cultural differences were found. Koreans were more likely to use SNS to increase contact but tended to spend less time browsing contacts' profiles than the Malaysians and Chinese. The intensity of SNS use differed between the countries as well, where Malaysians reported higher intensity than Koreans and Chinese. Consistent with study predictions, Koreans were found with the highest Autonomous-Related self-construal scores. The Autonomous-Related self-construal predicted SNS intensity. The findings suggest that cultural contexts, along with the way the self is construed in different cultures, may encourage different types of SNS usage. The authors discuss study implications and suggest future research directions.

## Introduction

Social Networking Sites (SNS) use and its implications for human behavior and emotion have received much attention from researchers in the past years. Early research which focused on the motives of SNS use indicated that the use of SNS is mainly motivated by social needs (e.g., Sheldon, 2008). Research in this area has expanded to focus on other psychological aspects, such as the users' well-being. Both positive and negative impacts on well-being have been reported (e.g., Pantic et al., 2012; Oh et al., 2013; Apaolaza et al., 2014). For instance, SNS use was found to be related to depressive symptoms, where heavy SNS use was correlated with depression severity (e.g., Pantic et al., 2012). SNS use was found to detrimentally impact romantic relationships (e.g., Elphinston and Noller, 2011). However, some research has illustrated the benefits of SNS usage, such as enhanced social capital and friendships of the users (Ellison et al., 2007, 2011; Steinfield et al., 2008; Valenzuela et al., 2009; Wang et al., 2014). Research has also indicated that SNS use may provide greater benefits to users with low self-esteem and low life satisfaction (Ellison et al., 2007; Steinfield et al., 2008). Personality research has been a key focus area within the SNS literature (e.g., Ong et al., 2011), while studies investigating the complicated interrelationships between sociocultural and individual level factors have been rarer to find.

Research on SNS has predominantly been carried out in Western, developed countries, where both development and testing of theories and measures have relied only on samples from such contexts. Existing cross-cultural studies, moreover, have reported on the differences between the Western, or the more individualistic, and the Eastern, more collectivistic countries, resulting in monolithic concepts of the prototypical Western or Eastern cultures, which overlook differences within each of these cultures, for example, amongst different Eastern countries (Kitayama et al., 2009). With behaviors that relate to interpersonal goals, especially, it may be important to look closely at whether similarities are observed in cultures that are supposed to be collectivistic, in order to come up with a more indigenous understanding of the behaviors and motivations in each of these cultures. We may find that the relative importance of the autonomy or relatedness, as well as behaviors on SNS with interpersonal goals may differ amongst these countries. For instance, if we were to adhere to Kagitcibasi's (2007) theory of social change, countries with similar levels of socioeconomic developments would also perceive the self and interpersonal distance similarly, which could mean that the difference between Western and Eastern countries may be smaller than one might expect, as long as they are both developed and economically stable. It may be that what we find with South Koreans in our study may be found with some Western countries that are at similar levels socioeconomically, whereas the difference between South Korea with the other two Asian countries, Malaysia and China may be larger because of the disparity in their levels of socioeconomic development. Moreover, given the strict government control in China, the intensity and the type of SNS use in China may also differ from the other two countries as well as other more liberal nations in the West. Therefore, instead of comparing Western versus Eastern differences on interpersonal behaviors on SNS, we decided to concentrate on investigating differences amongst three Asian countries, comparing between different motivations and the possible socioeconomic and political influences on SNS use in these countries. Despite the potential importance of considering the understanding of the self and its relation to close others and its implications for SNS usage, sociocultural influences on SNS usages, and especially self-construals, have been rarely studied (Tamam et al., 2009). Self-construals are perceptions of the self that take account of how the self relates to close others (Markus and Kitayama, 1991). Traditionally, individuals were deemed to emphasize either the independent/autonomous or the interdependent/relational aspect of the self. Individuals who emphasize the independent or autonomous self-construal often perceive themselves as unique and distinct from others, and thus, the expression of unique attributes and pursuit of their own goals are prioritized over others' needs (Markus and Kitayama, 1991; Singelis, 1994). Contrasting with the independent self-construal, the interdependent, or the relational self-construal, is a flexible self-construal that emphasizes connectedness with other individuals (Singelis, 1994). Often, individuals with this self-concept are more likely to behave according to the expectations from others instead of pursuing their own desires (Markus and Kitayama, 1991; Singelis, 1994).

Traditionally, these self-construals were considered to be incongruent, that the expression of one of the described self-construals indicated the absence of the other (e.g., Markus and Kitayama, 1991). However, Kagitcibasi (1996) proposed that these self-construals may coexist and that individuals may equally embrace and emphasize both of these self-construals simultaneously. This new model of conceiving the self, the Autonomous-Related self-construal, which refers to the balanced state of autonomous and relatedness may be seen as healthier, for it satisfies both needs where one can be independent and yet have the social and emotional support when needed. This theory of self was supported by previous findings which found individuals with high scores for both self-construals (e.g., Berry and Kim, 1988; Kim et al., 1996). In this study, we test the impact of this self-construal in predicting SNS intensity and type of social and interpersonal goals and behaviors on SNS. We believe that SNS may be a good medium for those who value both autonomy and relatedness, since they can choose autonomously how and when to interact with people, while engaging in these behaviors will allow them the sense of connectedness. We also tested for possible cultural differences across the Asian countries under investigation.

The link between SNS use and self-construals merits greater attention. Despite the importance of considering sociocultural influences on such behaviors, there is a scarcity of such research (Tamam et al., 2009). SNS use may relate differently in different cultures to social relationships, which could have positive or negative impacts on well-being. Moreover, efforts to foster healthy SNS use need a foundation of knowledge to be based upon and thus call for more cross-cultural investigations. In addition, problematically, results from previous studies have not led to consistent outcomes, possibly due to differences in their approaches. Although, researchers have mostly agreed that the sociocultural environment influences SNS usage via how it shapes the self, their findings have been in different directions. While some found a link between independent self-construal and SNS usage, others found a stronger link between interdependent self-construal and SNS usage (e.g., Kim et al., 2010; Chen and Marcus, 2012; Long and Zhang, 2014).

This study hypothesized that the Autonomous self-construal (Markus and Kitayama, 1991; Singelis, 1994) may positively predict general use of SNS, as measured by the intensity of SNS use. Those who perceive the self to be unique and separate from others may use SNS as a platform for promoting and reinforcing their self-image. Given that the related self-construal emphasizes relatedness (Markus and Kitayama, 1991; Singelis, 1994), it was hypothesized to have a positive relationship with the tendency to browse the contacts' profiles, which may be performed as a way to maintain social contact.

Therefore, we hypothesized that the Autonomous-Related self-construal, a balanced state of incorporating both autonomy and relatedness in how the self is constructed in relation to close others (Kagitcibasi, 1996, 2007), will be positively related to social and interpersonal behaviors on SNS measured in the present study. Contradicting with the traditional view of the selves, where both were deemed to be mutually exclusive (Markus and Kitayama, 1991), Kagitcibasi (1996, 2007) presented the Autonomous-Related self-construal as a new merged way of construing the self that may serve human needs better. Her theory is consistent with some previous findings, which revealed individuals having high scores for both self-construals (Berry and Kim, 1988; Kim et al., 1996), and can pave way for research that does not assume the simple East-West division.

For example, Levine et al. (2003) found that the related self-construal is not necessarily more dominant amongst the Asians. They observed that Americans scored significantly higher than Koreans for the related self-construal. Amongst the Koreans, Levine et al. (2003) found that the mean for the measured related self-construal was significantly lower than the mean for the measured Autonomous self-construal. These findings were deemed to support that individuals may incorporate features of the different selves into their self-system (Arnocky et al., 2007; DeCicco and Stroink, 2007), which helped justify the need for the balanced state of self in the present research. In addition, the Autonomous-Related self-construal was described as a self-concept which is consistent with socioeconomic development (Kagitcibasi, 2012, 2013). Hence, the Autonomous-Related self-construal suits the need for the present research that examines the use of SNS cross-culturally in countries with different socioeconomic development levels.

The most common way for measuring SNS use is by simple participant estimations in terms of the time (e.g., minutes or hours) spent on SNS or the frequency of use (e.g., Junco, 2012a,b). Despite its convenience, Anderson et al. (2012) listed the disadvantages for using such estimations, describing that inconsistent estimation may lead to different interpretations for the obtained results. In a related vein, McCord et al. (2014) argued that simply rating the amount of time spent on SNS could not reflect on the actual motivations for use. As these researchers have asserted the disadvantages for using simple estimations made by participants (Anderson et al., 2012; McCord et al., 2014), the present study measured SNS use with a standardized scale [i.e., the Facebook Intensity Scale (FIS)]. To reflect on general SNS use, the FIS (Ellison et al., 2007) was used. Additionally, the study innovatively measured several different aspects of SNS usage, aiming to tap into different possible motivations for use.

At the present, there are only a limited number of measurement tools for SNS usage that have been utilized and validated, especially with non-Western samples. Problematically, existing measures tend to have biases, testing only very specific aspects or ranges of SNS-related behaviors. Most measures were developed to measure for addictive tendencies toward SNS, such as the Bergen Facebook Addiction Scale (BFAS; Andreassen et al., 2012). Currently, the BFAS (Andreassen et al., 2012) is the measurement tool with the most adequate psychometric screening. However, it may not be suitable for studies which intend to measure normal, non-maladaptive SNS use, as for the present study that focuses on the interpersonal behaviors on SNS. In addition, measurement to gauge the intensity of specific activities on other users' profiles is not available; consequently, a scale that measures this construct will be composed as part of this study. As this construct is relatively new, the scale's psychometric properties will be examined. The measurement invariance for SNS scales has not been well examined in the literature; thus, the present study will address this gap by examining the equivalence of the construct across cultures.

One of the main functions for SNS use may involve achieving interpersonal goals. SNS has been suggested to promote online friendships which may facilitate real-world friendship quality, fostering users' wellbeing (Wang et al., 2014). Wang et al. (2014) explored in a Chinese university sample the influence on friendship quality and well-being of different types of SNS use, finding that participants who used SNS more frequently for social communication reported higher levels of well-being. These findings demonstrate that the use of SNS, especially in the Asian context, is linked closely to users' social relationships, which serves a beneficial role in their lives rather than to cause disturbances.

Managing existing contacts is a key social function that SNS serves (Boyd and Ellison, 2007), which sometimes can be portrayed by the exchange of information amongst the users (Kaplan and Haenlein, 2010). For the purpose of exchanging information, it is necessary for users to browse their contacts' profiles. This has been indicated in a research study, which stated that some users spent more time browsing other users' profiles than posting up relevant updates on their own SNS profiles (Pempek et al., 2009). This browsing behavior could be a consequence of the need for connectedness (e.g., Sheldon, 2008) and similar forms of behavior have been consistently described in previous research. This can be illustrated by Vasalou's et al. (2010) “Social browsing” subscale, which included an item to measure for the frequency of browsing other users' profiles. Similarly, Park and Kim's (2013) “Personal involvement” subscale also included a similar item, which measures for the tendency for checking other users' status and information on SNS. Further, evidence was provided by research that investigated stalking behavior on SNS. These research studies indicated that respondents browsed their contacts' profiles through activities like posting nasty comments on an ex-partner's photo (Lyndon et al., 2011) and reading an ex-partner's wall conversation (Chaulk and Jones, 2011). Further, Junco (2012a,b) listed a few activities including “Checking on friends,” which can be deemed as vaguely reflecting on the tendency to browse the contacts' profiles. However, the tendency to browse the contacts' profiles has not been adequately described in the past literature. Hence, a chasm in the literature has been introduced and the present research intends to examine the construct of SNS usage for such tendency. It was decided to compose a standardized scale to measure for the tendency to browse the contacts' profiles.

Cultural differences have been found in users' online behaviors, in research which involved comparisons between individualist and collectivist countries (e.g., Chu and Choi, 2010; Choi et al., 2011; Cho and Park, 2013; Jackson and Wang, 2013). These approaches applied the Individualism score from Hofstede's (2001) IBM survey, where certain countries were labeled as individualist and some were labeled as collectivist. This approach may have undermined the differences that existed in the individual level, as previous research found significant differences in countries with similar national culture (e.g., Kim and Leung, 2007). These suggest that there are other cultural and socioeconomic differences to consider. In this light, the present study that intends to investigate the relevance of the Autonomous-Related self considered the socioeconomic development of the countries, instead of the national culture proposed by Hofstede (2001). The Human Development Index, which is a composite formed by statistics of life expectancy, education, and income per capita, (HDI; United Nations Development Programme, 2013), often serves as an indicator of socioeconomic development. This index indicates that these countries are at different stages of socioeconomic development. Three Asian countries, namely Malaysia, China, and South Korea, were chosen for the present research to illuminate a cross-cultural comparison within Asian countries. In 2012, it was estimated that China had 597 million active SNS users (Go-Globe, 2013), followed by Korea with 24.8 million of users (Statista, n. d.), and Malaysia with 13.3 million of active users (Malaysia Asia, 2013). These countries all scored relatively low on the dimension of Individualism, suggesting that these countries were all collectivistic (Hofstede, 2001). However, previous research documented significant differences in samples with similar national culture (e.g., Kim and Leung, 2007). South Korea is far more developed than Malaysia and China. Malaysia, however, is more developed than China. Therefore, differences amongst the selected countries can be expected, despite similar scores for the dimension of Individualism (Hofstede, 2001).

Kagitcibasi (2012, 2013) had suggested, individuals develop a stronger sense of Autonomous-Related self in developed countries as they move toward more economic independence and yet maintain a continued sense of connectedness with their families. Thus, the present research intends to examine whether there are differences in the Autonomous-Related self-beliefs held by young adults in the three countries, as well as whether this self-construal has an association with SNS use. As indicated by the HDI (United Nations Development Programme, 2013), the socioeconomic development of South Korea exceeded Malaysia and China; thus, it was hypothesized that Koreans would have a stronger sense of Autonomous-Related self compared to the Malaysians and Chinese.

The present cross-cultural study aims to examine the role of Autonomous-Related self-construal in predicting different aspects of SNS usage in young adults from South Korea, China, and Malaysia. Cultural differences in self-construals and SNS usage as well as the linkages between the culture, self-construals and different types of behaviors on SNS will also be investigated. More specifically, this study takes a step forward from the existing literature looking more in depth at the intensity of SNS usage by focusing in more on the different SNS behaviors that are specific to connecting with others online and examining whether we can infer particular cultural influences on these SNS behaviors.

## Materials and methods

### Participants

A total of 305 undergraduate student participants were recruited. The age range was 18 to 28 and the mean age was 22.38 (*SD* = 3.27). Participants were recruited from Malaysia (*n* = 105), South Korea (*n* = 113), and China (*n* = 87). The Malaysian sample consisted of 28 (27%) male and 77 female (73%) participants with a mean age of 23.22 (*SD* = 2.59). The Korean sample comprised of 54 (48%) male and 59 (52%) female participants with a mean age of 22.27 (*SD* = 2.18). The Chinese sample consisted of 20 (23%) male and 67 (77%) female participants with a mean age of 20.70 (*SD* = 2.07). The participants were all recruited from medium-sized universities (with student numbers of <25,000) in capital cities of the respective countries. They were all thought likely to come from middle to middle upper class families, considering the high living costs in the capitals and the high tuition fee for tertiary education. Overall, the demographics for the participants were similar across the countries. Ethics approval was granted by the Monash University Human Research Ethics Committee.

### Measures

The questionnaire for the present study was translated into the native languages for respondents from Korea and China. Beaton's et al. (2000) translation-back translation method was used to ensure that the items were properly translated. The questionnaires were translated by native speakers with postgraduate qualifications in social sciences subjects, and the translated versions were back-translated into English to ensure that the content and the meanings of the items remained the same. For the Malaysian sample, the questionnaire was administered in English, as participants were recruited from an international university, where fluency in English is compulsory for admission.

The rating scales in the present questionnaires were altered to not contain neutral options, since past research has suggested that participants from collectivistic cultures tend to select the middle response (e.g., Bennett, 1977; Harzing, 2006). All the questionnaire items contained 6-point rating scales, ranging from 1 (*strongly disagree*) to 6 (*strongly agree*).

It is necessary to reassess the model fit for the scales as the ratings were modified. Hence, each of the respective scales was subjected to CFA. The model fit for the models was evaluated using cutoff criteria recommended by Hu and Bentler (1998; RMSEA ≤ 0.06, CFI ≥ 0.95, and SRMR ≤ 0.08). MacCallum et al. (1996) noted that a RMSEA ranging from 0.08 to 0.10 indicates a mediocre fit. Therefore, for the present CFA, the RMSEA ranging from 0.08 to 0.10 will still be accepted, while the ideal is 0.06 (Hu and Bentler, 1998). The model fit will be assumed as long as the values for the imposed cutoff were met since the chi-square is sensitive to sample size, where significance may not reflect on misfit of the model (Blunch, 2008). Multi-group confirmatory factor analysis was performed on the scales as well, constraining the model to be equal across the countries. This is intended to ensure that the scales have similar construct validity across the countries. To test differences amongst the groups, the restricted chi-square test (Δχ^2^) was calculated with the alpha level of 0.01.

The demographic variables assessed included age, gender, and nationality of the participants. Additionally, four questionnaires were completed, including the Activities on Others' Profiles Checklist (AOPC) and the Contact with Friends' Profiles (CFP), which were developed by the authors.

The intensity of SNS use was measured with the FIS (Ellison et al., 2007), which consists of eight items. For the present study, the items were rephrased to suit looking at SNS usage in general, instead of solely on Facebook. A sample item is “I am proud to tell people I am on social networking sites.” Initially, the scale developed by Ellison et al. (2007) consisted of a 5-point rating. For the purpose of controlling the tendency to select the middle response, the rating scale was converted into a 6-point scale in this study. The model fit was achieved for each country [Malaysia: $\chi_{\left(17\right)}^{2}$ = 17.20, *p* = 0.44, RMSEA = 0.01 (CI = 0.00–0.09), CFI = 0.99, SRMR = 0.04; Korea: $\chi_{\left(19\right)}^{2}$ = 32.63, *p* < 0.05, RMSEA = 0.08 (CI = 0.02–0.12), CFI = 0.97, SRMR = 0.04; China: $\chi_{\left(18\right)}^{2}$ = 27.07, *p* = 0.07, RMSEA = 0.07 (CI = 0.00–0.13), CFI = 0.98, SRMR = 0.05]. The model was constrained to be equal for each country, and the change in the chi-square was significant, indicating that some items were interpreted differently. These were the items that assessed for the amount of time, the number of friends on SNS, emotional aspect of SNS use and the frequency of SNS use. Removal of these items reduced the fit indices, and thus, the constraints for these items were relaxed, allowing them to differ across cultures. Eventually, the change in the chi-square was not significant, Δ$\chi_{\left(8\right)}^{2}$ = 11.63, *p* = 0.16. The Cronbach's alphas were all within the acceptable range (Malaysia = 0.86; Korea = 0.90; China = 0.87).

To measure the tendency for browsing the contacts' profiles, a new scale was developed. The initial item pool for the Activities on Others' Profiles Checklist (AOPC) consisted of 26 items, which reflect on different activities on SNS that are to do with checking or engaging other users' profiles. The items were obtained from the results of a pilot study that was conducted prior, where 20 university student SNS users (9 males and 11 females) were requested to list 10 different types of activities they conduct on their contacts' profiles. These activities were pooled together to form the AOPC and the 26 selected items were then administered to the participants of this current study. The items were rated on a 6-point scale, ranging from 1 (*strongly disagree*) to 6 (*strongly agree*). From the exploratory factor analysis, 17 items were found with a loading below 0.30. Thus, these items were removed. The 9 items were found with KMO of 0.87, and the Bartlett's test of sphericity was significant, $\chi_{\left(36\right)}^{2}$ = 1295.20, *p* < 0.001. The two components explained for 50.15 and 17.16% of the variance, respectively. The eigenvalues for the components were 4.51 and 1.54, respectively. The decision to retain two components was supported by the parallel analysis as well. The first component was named as Increasing Contact, while the second component was named as Updating. The model was found with good fit for each country [Malaysia: $\chi_{\left(26\right)}^{2}$ = 38.30, *p* = 0.05, RMSEA = 0.06 (CI = 0.00–0.11), CFI = 0.96, SRMR = 0.05; Korea: $\chi_{\left(26\right)}^{2}$ = 44.67, *p* = 0.01, RMSEA = 0.08 (CI = 0.03–0.11), CFI = 0.95, SRMR = 0.06; China: $\chi_{\left(23\right)}^{2}$ = 34.35, *p* = 0.06, RMSEA = 0.07 (CI = 0.00–0.12), CFI = 0.98, SRMR = 0.04]. The model was constrained to be equal for each of the countries and the change in the chi-square was significant, Δ$\chi_{\left(14\right)}^{2}$ = 36.92, *p* = 0.001. A few constraints on the factor loadings were relaxed and the change in the chi-square was not significant, Δ$\chi_{\left(6\right)}^{2}$ = 4.49, *p* = 0.61. The Cronbach's alphas for the Increasing Contact component (Malaysia = 0.81; Korea = 0.79; China = 0.94) and the Updating component (Malaysia = 0.86; Korea = 0.82; China = 0.87) were all satisfactory. High score for the Increasing Contact component indicates for a higher tendency to browse the contacts' profiles for the purpose of increasing the amount of interaction with the respective contacts. A high score for the Updating component indicates for the higher tendency to browse the contacts' profiles for the purpose of gaining updates from the contacts. Table 1 summarizes the factor loadings for each respective country.

**Table 1:** **Factor loadings of AOPC**.

**Items**	**Malaysia**		**Korea**		**China**	
	**Increasing contact**	**Updating**	**Increasing contact**	**Updating**	**Increasing contact**	**Updating**
Share events	0.77		0.67		0.83	
Invitation to group chat	0.76		0.63		0.93	
Invitation to join event	0.67		0.68		0.91	
Send game request	0.61		0.65		0.81	
Initiate video call	0.58		0.68		0.86	
View post from contacts' profile		0.96		0.61		0.91
Check for updates		0.84		0.75		0.71
View statuses		0.72		0.82		0.81
View personal details		0.62		0.76		0.74

The tendency for browsing the contacts' profiles in general was measured by the Contact with Friends' Profiles (CFP) consisting of five items, which was designed for the present research. Although, the items appeared to be similar to those from the AOPC, the function of these items were relatively unspecified and more generally tapping on the social interactions on SNS. AOPC items are more detailed in their aims, in that those were directly associated with checking and other more pro-active behaviors. The items on this scale were also rated on a 6-point scale, ranging from 1 (*strongly disagree*) to 6 (*strongly agree*). For the EFA, the KMO was 0.81 and the Bartlett's test of sphericity was significant, $\chi_{\left(10\right)}^{2}$ = 375.40, *p* < 0.001. By default, a single component was identified, explaining for 53.47% of the variance. The eigenvalue for the component found was 2.67. The parallel analysis supported for the decision to retain the component. The model was found with good fit for each country [Malaysia: $\chi_{\left(6\right)}^{2}$ = 6.95, *p* = 0.32, RMSEA = 0.03 (CI = 0.00–0.13), CFI = 0.98, SRMR = 0.06; Korea: $\chi_{\left(5\right)}^{2}$ = 6.28, *p* = 0.27, RMSEA = 0.04 (CI = 0.00–0.14), CFI = 0.99, SRMR = 0.03; China: $\chi_{\left(4\right)}^{2}$ = 6.88, *p* = 0.14, RMSEA = 0.09 (CI = 0.00–0.20), CFI = 0.98, SRMR = 0.03]. The model was constrained to be equal for each country and the change in the chi-square was not significant, Δ$\chi_{\left(8\right)}^{2}$ = 17.97, *p* = 0.02. Overall, the Cronbach's alphas were all satisfactory (Malaysia = 0.65; Korea = 0.75; China = 0.87). Table 2 summarizes the factor loadings for each respective country.

**Table 2:** **Factor loadings of CFP**.

**Items**	**Malaysia**	**Korea**	**China**
Post comments	0.61	0.65	0.65
Initiate conversation	0.79	0.79	0.78
Tag	0.46	0.51	0.89
Reply messages	0.32	0.48	0.70
Invite to join group	0.42	0.63	0.66

Self-construals were measured with the Autonomy-Relatedness Scales (Kagitcibasi, 2007), which consists of 27 items. It has three subscales, each representing a different self-construal, namely the Autonomous self, Related self, and the Autonomous-Related self. Sample items for these subscales include “People who are close to me have little influence on my decisions” (Autonomous self), “I need the support of persons to whom I feel very close to” (Related self), and “Even if the suggestions of those who are close are considered, the last decision should be one's own” (Autonomous-Related self). This scale was originally rated on a 7-point scale, but in the present study the items were rated on a 6-point scale. Four items were removed from the original 9 item Autonomous-Related self-subscale. The Autonomous-Related self subscale with five items exhibited good fit for each country [Malaysia: $\chi_{\left(5\right)}^{2}$ = 5.47, *p* = 0.36, RMSEA = 0.03 (CI = 0.00–0.14), CFI = 0.99, SRMR = 0.03; Korea: $\chi_{\left(3\right)}^{2}$ = 4.64, *p* < 0.001, RMSEA = 0.07 (CI = 0.00–0.18), CFI = 0.97, SRMR = 0.04; China: $\chi_{\left(3\right)}^{2}$ = 4.65, *p* = 0.19, RMSEA = 0.08 (CI = 0.00–0.21), CFI = 0.98, SRMR = 0.03]. The model was constrained to be equal across the three countries and the change in the chi-square was not significant, Δ$\chi_{\left(8\right)}^{2}$ = 11.08, *p*=0.19. The Cronbach's alphas for the Autonomous-Related self-construal scale were satisfactory (Malaysia = 0.76; Korea = 0.62; China = 0.79).

## Results

### Data screening

Given that the components for the AOPC were decided, and the invariance of the factor loadings for each scale was supported by the analysis, the variables were computed according to the CFA. Statistical tests performed on these variables included hierarchical regression and ANOVA. The hierarchical regression intended to assess the significance of the self-construal in predicting for the SNS usages, while controlling for demographics variables, such as age and gender. The ANOVA intended to test for country differences in SNS usages and self-construal. Initially, the variables were screened for univariate and multivariate outliers. Outliers were identified with (1) *z*-score larger than 3.29, and (2) a *p* < 0.001 for Mahalanobis distance and Cook's distance larger than 1.00 (Tabachnick and Fidell, 2012). One univariate outlier for the Autonomous-Related self was identified, with the *z* = −3.89. It was transformed to the next largest value, which was −2.40. There were no multivariate outliers identified.

The distributions for the variables were assessed with both visual inspection and the Kolmogorov-Smirnov (KS) test. The KS tests were all significant, indicating that the distributions were skewed. However, visual inspections revealed that the distributions were all relatively normal, except for the Increasing Contact subscale. Log and square root transformations were performed, and the same visual inspection did not reveal any improvement in the skewness. However, there were at least 20 samples in each cell, which is sufficient to ensure robustness (Tabachnick and Fidell, 2012). The Levene's tests indicated that the variances were not homogenous. It was recommended to use a more stringent alpha value (Tabachnick and Fidell, 2012), and thus, the present alpha value was set at 0.01. After screening for the basic assumptions, the descriptive statistics were summarized in Table 3.

**Table 3:** **Descriptive statistics**.

**Variables**	**Malaysia**	**Korea**	**China**
Autonomous-related self	4.64 (0.87)	5.59 (0.51)	4.60 (0.80)
Browsing contacts' profiles	2.76 (0.82)	2.35 (0.85)	2.75 (1.03)
Increasing contact	1.94 (0.84)	2.82 (1.04)	2.36 (1.26)
Updating	3.15 (1.13)	3.42 (1.14)	3.22 (1.25)
Intensity of SNS use	4.04 (1.04)	2.72 (1.13)	3.45 (0.99)

*Figures in the brackets are the standard deviations*.

### Country comparisons for SNS usages and self-construal

The test of ANOVA with α = 0.01 was conducted to examine for country differences. The stringent alpha value was employed since there were violations of the assumptions for ANOVA (Tabachnick and Fidell, 2012). Moreover, multiple testing will increase the likelihood for committing type 1 error, which can be addressed with a stringent alpha value. The Tukey's test was used as the *post-hoc* test.

For the SNS intensity, the ANOVA was significant, with the *F*_(2, 302)_ = 41.69, *p* < 0.001, η = 0.21. The mean from the Malaysian sample was significantly higher than the Korean sample, *p* < 0.001 and large effect size of *d* = 1.21, and the Chinese sample, *p* < 0.001 and medium effect size of *d* = 0.58. The difference between the Chinese and the Korean samples was also significant, *p* < 0.001 and medium effect size of *d* = 0.68.

For the Increasing Contact subscale, the ANOVA revealed significant differences, with the *F*_(2, 302)_ = 18.95, *p* < 0.001, η = 0.11. The mean from the Malaysian sample was significantly lower than the Korean sample, *p* < 0.001 and small effect size of *d* = 0.42. The mean from the Chinese sample did not differ significantly from Malaysian sample, *p* = 0.02 and small effect size of *d* = 0.39, while it differed significantly from the Korean samples, *p* = 0.007 and small effect size of *d* = 0.39.

The ANOVA was not significant when the mean differences for the Updating subscale was assessed, *F*_(2, 302)_ = 1.57, *p* = 0.20, η = 0.01. However, the ANOVA which examined the mean differences for the CFP scale was significant, *F*_(2, 302)_ = 7.20, *p* = 0.001, η = 0.04. The Malaysians, *p* = 0.002 and small effect size of *d* = 0.49, and the Chinese, *p* = 0.006 and small effect size of *d* = 0.42, exhibited significantly higher mean than the Koreans for the general tendency to browse the contacts' profiles. The difference between the Malaysians and the Chinese, however, was not significant, *p* = 0.99 and *d* = 0.01.

The ANOVA revealed significant differences for the Autonomous-Related self, *F*_(2, 302)_ = 48.16, *p* < 0.001, η = 0.24. The mean from Korean sample was significantly higher than the means from Malaysian, *p* < 0.001 and large effect size of *d* = 1.19, and Chinese samples, *p* < 0.001 and large effect size of *d* = 1.32. The difference between the Malaysian and Chinese sample, however, was not significant, *p* = 0.94 and *d* = 0.04.

### Predicting SNS usages with self-construal

Hierarchical regressions were conducted for each country separately. The participants' age and gender were entered into the first block, while the self-construal was entered into the subsequent block. Participants' gender was dummy coded (0 = male, 1 = female) before the analysis. Table 4 summarizes the hierarchical regression for predicting SNS intensity with self-construal.

**Table 4:** **Hierarchical regression predicting for SNS intensity with self-construal**.

**Variables**	**Malaysia**		**Korea**		**China**	
	**First step**	**Second step**	**First step**	**Second step**	**First step**	**Second step**
Age	−0.07	−0.04	−0.22^*^	−0.25^*^	−0.10	−0.04
Gender	−0.12	−0.16	−0.12	−0.14	0.11	0.14
Autonomous-related self		0.22^*^		−0.19^*^		0.17
	*F*_(2, 101)_ = 1.11 *R*^2^ = 0.02	*F*_(3, 100)_ = 2.42 *R*^2^ = 0.06 *R*^2^ change = 0.04^*^	*F*_(2, 110)_ = 2.64 *R*^2^ = 0.04	*F*_(3, 109)_ = 3.24^**^ R^2^ = 0.08 *R*^2^ change = 0.04*	*F*_(2, 83)_ = 1.46 *R*^2^ = 0.03	*F*_(3, 82)_ = 1.82 *R*^2^ = 0.06 *R*^2^ change = 0.03

* p < 0.05,

** *p < 0.01*.

From Table 4, it was revealed that for the Malaysians and the Chinese, none of the demographic predictors significantly predicted for the intensity of SNS use. However, for the Malaysians and the Koreans, the Autonomous-Related self significantly predicted for the intensity of SNS use. For the Malaysians, the self-construal positively predicted for the intensity of SNS use. The Koreans revealed a contradicting result, where the same self-construal predicted for the intensity of SNS use negatively.

The hierarchical regression was conducted with the tendency for browsing the contacts' profiles in general as the criterion variable, with the similar variables in the first and second steps. Table 5 summarizes the hierarchical regression. From Table 5, the self-construal did not significantly predict for the tendency for browsing the contacts' profiles in general.

**Table 5:** **Hierarchical regression predicting for the tendency for browsing the contacts' profiles in general with self-construal**.

**Variables**	**Malaysia**		**Korea**		**China**	
	**First step**	**Second step**	**First step**	**Second step**	**First step**	**Second step**
Age	−0.06	−0.06	−0.15	−0.16	0.05	0.07
Gender	−0.26^**^	−0.27^**^	−0.18	−0.20^*^	0.04	0.05
Autonomous-related self		0.03		−0.12		0.08
	*F*_(2, 101)_ = 4.23^*^ R^2^ = 0.07	*F*_(3, 100)_ = 2.83^*^ R^2^ = 0.08 *R*^2^ change = 0.01	*F*_(2, 110)_ = 2.09 *R*^2^ = 0.03	*F*_(3, 109)_ = 1.98 *R*^2^ = 0.05 *R*^2^ change = 0.02	*F*_(2, 83)_ = 0.12 *R*^2^ = 0.003	*F*_(3, 82)_ = 0.23 *R*^2^ = 0.009 *R*^2^ change = 0.006

* p < 0.05,

** *p < 0.01*.

The subscales for the AOPC were entered into the regression model as the criterion variables, with similar variables in the first and second steps. Table 6 summarizes the hierarchical regression, predicting for the tendency for browsing the contacts' profiles for increasing the amount of interaction. Table 6 illustrates that the self-construal did not significantly predict for such a tendency.

**Table 6:** **Hierarchical regression predicting for the tendency for browsing the contacts' profiles for increasing the amount of interaction with self-construal**.

**Variables**	**Malaysia**		**Korea**		**China**	
	**First step**	**Second step**	**First step**	**Second step**	**First step**	**Second step**
Age	0.04	0.01	−0.14	−0.13	0.30^*^	0.34^*^
Gender	−0.16	−0.13	−0.16	−0.15	0.06	0.08
Autonomous-related self		−0.19		0.11		0.13
	*F*_(2, 101)_ = 1.54 *R*^2^ = 0.03	*F*_(3, 100)_ = 2.33 *R*^2^ = 0.06 *R*^2^ change = 0.03	*F*_(2, 110)_ = 1.68 *R*^2^ = 0.03	*F*_(3, 109)_ = 1.60 *R*^2^ = 0.04 *R*^2^ change = 0.01	*F*_(2, 83)_ = 3.78^*^ R^2^ = 0.08	*F*_(3, 82)_ = 3.04^*^ R^2^ = 0.10 *R*^2^ change = 0.02

* *p < 0.05*.

The hierarchical regression was repeated with the tendency for browsing the contacts' profiles for the purpose of gaining updates as the criterion variable, with the same variables for the first and second steps. Table 7 summarizes the hierarchical regression. As shown in Table 7, the self-construal did not significantly predict for such a tendency.

**Table 7:** **Hierarchical regression predicting for the tendency for browsing the contacts' profiles to gain updates with self-construal**.

**Variables**	**Malaysia**		**Korea**		**China**	
	**First step**	**Second step**	**First step**	**Second step**	**First step**	**Second step**
Age	0.01	0.04	−0.14	−0.12	−0.04	−0.01
Gender	−0.14	−0.17	−0.03	−0.01	0.05	0.06
Autonomous-related self		0.18		0.16		0.09
	*F*_(2, 101)_ = 1.03 *R*^2^ =0.02	*F*_(3, 100)_ = 1.78 *R*^2^ = 0.05 *R*^2^ change = 0.03	*F*_(2, 110)_ = 0.99 *R*^2^ = 0.02	*F*_(3, 109)_ = 1.65 *R*^2^ = 0.04 *R*^2^ change = 0.02	*F*_(2, 83)_ = 0.19 *R*^2^ = 0.005	*F*_(3, 82)_ = 0.33 *R*^2^ = 0.012 *R*^2^ change = 0.007

## Discussion

A central aim of the present study was to explore the relationship between the Autonomous-Related self-construal and various aspects of SNS usage, which we hypothesized, had some cultural implications. Overall, cross-cultural differences were observed in the endorsement of the self-construal and some of the aspects of SNS use measured. The self-construal significantly predicted for the intensity of SNS use for Malaysians positively and Koreans negatively. However, the self-construal did not significantly predict for other aspects of SNS use.

### The present measurements

For the newly devised scale on the tendency to browse the contacts' profiles (AOCP), the EFA revealed that the items loaded on the respective components without overlapping, which supported the proposed structure (Thurstone, 1947). This finding supported the clarity of the two factors found, which were Increasing Contact and Updating, which marks the strength of the present scale. These newly developed scales can be used in future studies to measure for the different aspects of SNS use, which have not been explored in-depth in the past.

The CFA performed supported the model fit of the newly devised CFP and the AOPC, where the items were interpreted similarly across the cultural groups. Such consistency can be partly attributed to the simplicity of language used in the items for the scale and demonstrates that translation errors are unlikely, accounting partly for the invariant factor loadings (Cheung and Rensvold, 2002). The Cronbach's alphas indicate good agreement for the items which form the scales. The results from the confirmatory factor analysis performed support that the forced response scaling did not alter significantly the factor structures of the scales.

Four items on the SNS intensity scale (SIS), amount of time spent on SNS, frequency of use per day, number of friends on SNS and the emotional aspects of SNS use, differed across the countries. This cross-cultural observation is consistent with past studies, which similarly revealed differences across countries (Chu and Choi, 2010; Choi et al., 2011; Jackson and Wang, 2013). In addition, a past study also illustrated differences in the ratings for the importance of SNS across different cultures (Jackson and Wang, 2013), which included pride as one of the aspects for importance and suggests that pride for being a SNS user may differ across cultures. The reviewed findings suggest that the variant loadings found were secondary to cultural differences. The present results suggest that the items for the original SIS, the altered Facebook Intensity scale, require amendments to address better these differences. Overall, these differences support a call for more cross-cultural investigations, as well as a stronger focus on sociocultural impacts on such behaviors', which have not yet been examined in detail in past studies.

The CFA revealed a poor fit for the Autonomous-Related self-construal, which was assessed initially with the nine items from the Autonomous-Relatedness scale (Kagitcibasi, 2007). Four items reduced the Cronbach's alpha, and with these items removed from further analysis, the remaining five items indicated good fit indices. The problem with the items may be due to the negative wording of these items, which several researchers have cautioned against their inclusion in developing a scale (e.g., Peterson et al., 2006; Roszkowski and Soven, 2009). A study conducted by Merritt (2012) found that fatigued participants may lack the cognitive resources to comprehend survey items which are negatively worded. With the removal of the items, the factor structure was equivalent across the countries with the remaining items, which suggests that it may be necessary to revise the items for the Autonomous-Related self.

### Cross-cultural differences in SNS usages and self-construal

Results from the cross-cultural comparison revealed that the Malaysians had significantly higher self-reported SNS intensity than the Koreans and the Chinese. In addition, the Chinese sample mean was significantly higher than that of the Korean sample. These findings indicate that the Malaysian sample may use SNS in a more intense manner than the Chinese and the Koreans. This difference could be due to the different experiences they may have with SNS, as well as the freedom and the nature of shared communications on SNS. One potential difference that may be highlighted, for instance, is the presence of the Great Firewall of China, which altered the experience of SNS use with its heavy filtering (Qin, 2011).

Unlike their Chinese counterparts, the Koreans were not exposed to such strict monitoring (Chung, 2008). Although the Korean authorities do exercise some control over the internet use, it was not severe to the extent that it will alter the experience of SNS use. This suggests that there must be other explanations for the low intensity of SNS use amongst the Koreans. A qualitative study conducted with Americans and Koreans may provide a partial explanation for the present results (Cho and Park, 2013), as the authors observed a recent trend in Korea, where SNS users tend to unsubscribe from SNS to remove acquaintances from their SNS pages (Cho and Park, 2013). This act was motivated by the users' strong need to maintain their relationships with those whom they are close to and not be distracted by online social networking. This possibility is supported by Kim's et al. (2011) results which indicate that the Koreans relied heavily on their existing and long-lasting contacts for social support and information. Due to their reliance on their existing contacts, it is necessary for the Koreans to maintain their connection with these users, which eventually leads to the drastic measure of unsubscribing from their SNS to filter out acquaintances. Due to such a tendency, the Koreans' SNS intensity may have been reduced, accounting for the differences found by the present study. These results highlight the importance of the sociocultural influences on SNS use and the shaping of behaviors, which need to be considered more carefully in future research.

The present results revealed that cross-cultural differences in the tendency to browse the contacts' profile for updating purposes were not significant. This non-significant finding suggests that such a tendency was equal across the three cultural groups. In addition, such behavior can be considered as common amongst SNS users as the main likely reason for their use is to maintain connections with other users. Various studies have indicated that another purpose for SNS use is to seek for information (Chang and Zhu, 2011; Kim et al., 2011). The present results also revealed that the Koreans were more likely to use SNS to increase contact with their SNS contacts than the Malaysian and the Chinese. This finding is consistent with Kim's et al. (2011) findings, that the Koreans were more reliant on their contacts for support, which motivated them to increase contact with their connections on SNS. Moreover, contacts for the Korean users have tended to be close friends (Cho and Park, 2013). Therefore, it is reasonable that they used SNS to increase the amount of interaction with the other users. The findings from our study also indicate that the general tendency for browsing the contacts' profiles for the Koreans was less than the Malaysians and the Chinese. This observation may be accounted for by the nature of the relationship pursued by the Koreans. It was indicated that the Koreans emphasized on certain contacts whom they are closely connected to (Cho and Park, 2013). Therefore, their use of SNS is directed to these contacts; their use of SNS is more specific. Our findings were significant but with small differences between the groups. This may imply that the measured behaviors on SNS have different variations, which were not accounted by the present measurements. This could also indicate that users from these countries had adapted their behavior on SNS in a coherent fashion (Chu and Choi, 2010). Therefore, there was only slight discrepancy in the displayed behavior on SNS. For the Autonomous-Related self-construal, the Koreans scored significantly higher than the Malaysians and the Chinese, suggesting that the Koreans have a greater balanced state of both autonomy and relatedness than the Malaysians and the Chinese. Results indicated that the Autonomous-Related self-construal is more commonly found in Korea, a country with greater socioeconomic development than Malaysia and China (United Nations Development Programme, 2013), and thus, supported Kagitcibasi's (2012, 2013) theory. Our findings do seem to indicate that the more developed and stable the country is, the more likely it is that its citizens will place emphasis equally on both autonomy and relatedness of the self. Similarity in the scores of the Malaysian and the Chinese samples can thus also be explained by the difference in the socioeconomic conditions between the two countries. Consistent with the similar rankings for the development index consulted (United Nations Development Programme, 2013), the difference for the Autonomous-Related self-construal between the Malaysians and the Chinese was not significant.

### Self-construal in predicting behaviors on SNS

The present study found that the Autonomous-Related self-construal significantly predicted for the intensity of SNS use amongst the Koreans and the Malaysians. However, even for these two countries, the direction of the predicted relationship between Autonomous-Related self-construal and the intensity of SNS use differed across the countries. It was found that the Autonomous-Related self-construal predicted for the Malaysians' intensity of SNS use positively, while for the Koreans it predicted intensity of SNS use negatively, which may indicate a pattern of cross-cultural difference. From the present findings, it was suggested that for the Malaysians, the balanced state between autonomy and relatedness predicted for higher intensity of SNS use. The intensity of SNS use for the Malaysians may have been driven equally by both self-construal aspects, autonomy, and relatedness. However, for the Koreans, it was indicated that the intensity of SNS use was predicted by the low balanced state between autonomy and relatedness. This finding suggests that for the Koreans, one of the self-aspects predominantly predicted for the intensity of SNS use. Further, examination on this aspect was not conducted in the present study, since the scales measuring for the autonomous and relatedness aspects failed to achieve the basic model fit, indicating that the interpretation for the constructs may not be meaningful. This area requires attention from future researchers to include the different aspects of self into consideration. It is also recommended for future research to further validate the self-construal scales cross-culturally.

However, the Autonomous-Related self-construal failed to predict other SNS related behaviors. Despite, its significance in predicting for the intensity of SNS use, the Autonomous-Related self-construal was found with low *R*^2^ which indicated for low predictive power for SNS related behaviors, suggesting that there are other factors which are more influential in predicting for the participants' SNS use. The self-construal measure used in the present study did not substantially improve the predictive model, which may be accounted for by the scale's limitations or the scale's overall scope that may fail to capture specific behaviors and intentions such as the SNS related behaviors studied here.

### Implications

The present research adds to the literature on cross-cultural patterns of SNS use. At the present, most research has focused on the differences between individualist and collectivist cultures, such as America and China (e.g., Jackson and Wang, 2013). Additionally, the present research could be the first attempt to understand the role of Autonomous-Related self-construal on SNS use. The statistical significant differences found in the present study indicate that differences do exist amongst the collectivist countries. However, the differences found in the different aspects of SNS usage were consistently low. Thus, the differences should be interpreted cautiously. The low *R*^2^ values indicate that other factors, such as socioeconomic factors, may affect the use of SNS significantly. Results indicated that the mean for the Autonomous-Related self-construal for the Korean sample was significantly higher than the Malaysian and Chinese samples, at a huge margin. This result is consistent with the theoretical concept of the Autonomous-Related self-construal, which was described to be more prevalent in developed countries (Kagitcibasi, 2012, 2013).

In considering what may have been found if we included Western samples in our study, we believe that the interpersonal goals related to SNS use in Western countries may have differed significantly from the three Asian countries included here. Those from the West may still value autonomy more strongly than Asians, and in that sense maintaining interpersonal relations online may not be such a strong goal for them compared to Asians. However, the actual differences that may be found between Western and Eastern countries remain uncertain and our results do not extend to provide a clear answer to such a question. Similarly to our findings, differences amongst the Western cultures would also have been found depending on sociocultural factors such as the ones we discussed in the current paper and more research is needed that will allow for a more systematic cross-cultural comparison.

Our findings also suggest that SNS users do invest efforts in their contacts' profiles. The present EFA revealed two components, which were Updating and Increasing Contact. Future research can consider to further examine these aspects of SNS use. The need to establish measurement invariance should be stressed, to ensure that the variables compared are perceived similarly across cultures. This effort was present in the current investigation, and future research should replicate similar effort in the measurements used. From the multi-group CFA, it was revealed that certain items from the FIS (Ellison et al., 2007), which was presently adapted into the SIS, were interpreted differently across the cultural groups. As mentioned in the previous paragraphs, the differences found can be expected, supported by various research which conducted cross-cultural comparisons in the use of SNS (Chu and Choi, 2010; Choi et al., 2011; Jackson and Wang, 2013). The variant items suggest the need to revise the measure, or perhaps to compose a new measurement for SNS use.

### Limitations

The present study has a few notable limitations. Firstly, the present self-construal findings were limited to the Autonomous-Related self-construal. It remains as a question whether the dichotomized self-construals are able to account for more variance in predicting for SNS related aspects and future research is recommended to explore this possibility. Additionally, participants were limited to those from three collectivistic countries and future research is suggested to explore comparisons with participants from individualistic countries as well. As a result, the Autonomous-Related self-construal, which is less commonly focused upon in the self-construal literature, was the sole self-construal for the present examination. Caution is recommended in interpretation of the preliminary factor analysis. Future efforts to further examine the psychometric properties for these scales are highly recommended. Secondly, the present research relied heavily on self-reported data, and accuracy may have been reduced due to the need for social desirability or response biases. Future research should attempt to use other methods for assessing SNS use, such as utilizing the monitoring software employed by Junco (2013). Thirdly, the use of student sample limits the generalizability of the results. Moreover, the sample sizes of the present study can be considered small. These factors, altogether, may affect the outcomes of this study.

## Author contributions

All authors listed, have made substantial, direct and intellectual contribution to the work, and approved it for publication.

### Conflict of interest statement

The authors declare that the research was conducted in the absence of any commercial or financial relationships that could be construed as a potential conflict of interest.
